# Curved vs. Straight-Line Handwriting Effects on Word Recognition in Typical and Dyslexic Readers Across Chinese and English

**DOI:** 10.3389/fpsyg.2021.745300

**Published:** 2021-10-28

**Authors:** Connie Qun Guan, Yifei Li, Wanjin Meng, Laura M. Morett

**Affiliations:** ^1^Faculty of Foreign Studies, Beijing Language and Culture University, Beijing, China; ^2^School of Foreign Studies, Beijing University of Science and Technology, Beijing, China; ^3^Institute of Moral Education, Psychology and Special Education, China National Institute of Education Sciences, Beijing, China; ^4^Department of Educational Studies in Psychology, Research Methodology, and Counseling University of Alabama, Tuscaloosa, AL, United States

**Keywords:** handwriting, drawing, visual word recognition, N170, laterality, Chinese, English

## Abstract

Handwriting serves to link auditory and motor routines with visual word processing, which is a hallmark of successful reading. The current study aims to explore the effect of multisensory integration as a pathway to neural specialization for print among typical and dyslexic readers across writing systems. We identified 9–10-year-old dyslexic Chinese children (*n* = 24) and their typically developing counterparts (*n* = 24) on whom we conducted both behavioral and electroencephalogram (EEG) experiments. We designed four learning conditions: Handwriting Chinese (HC), Viewing Chinese (VC), Drawing followed by Character Recognition in Chinese (D-C), and Drawing followed by Word Recognition in English (D-E). In both handwriting and drawing conditions, we also designed curved vs. straight-line stimuli. Both behavioral and EEG results showed that handwriting straight line strokes facilitated visual word recognition in Chinese compared to handwriting curved lines. Handwriting conditions resulted in a lateralization of the N170 in typical readers, but not the dyslexic readers. Interestingly, drawing curved lines facilitate word recognition in English among dyslexic readers. Taken together, the results of the study suggest benefits of handwriting on the neural processing and behavioral performance in response to Chinese character recognition and curved-line drawing effects on English word recognition among dyslexic readers. But the lack of handwriting effects in dyslexic readers suggest that students who have deficits in reading may also be missing the link between multisensory integration and word recognition in the visual word form areas. The current study results have implications for maintaining handwriting practices to promote perception and motor integration for visual word form area development for normal readers and suggest that drawing practices might benefit Chinese dyslexic readers in reading English.

## Introduction

Writing meaningful symbols is a major landmark in the evolution of human culture. Handwriting connects visual word processing, a milestone for successful reading, with motor and auditory routines (Dehaene and Cohen, 2011). Early processing of visual word forms is constrained by the interaction with auditory and motor regions (Sekiyama et al., 2003; Wuerger et al., 2012; Callan et al., 2014), but the movement of handwriting promotes the integration of visual word forms through motor and auditory routines (Longcamp et al., 2006; Guan et al., 2011, 2021; James, 2017). Even though handwriting seems crucial for reading development, 30–50% of children with dyslexia show significant handwriting difficulties (Montgomery, 2008; Di Brina et al., 2018). These difficulties persist in college-age students and could possibly be associated with other sensory-motor integrative skills, like drawing (Sumner et al., 2014). The nature of the effect of handwriting on word recognition in students with dyslexia is still unclear, and most of our knowledge on this topic is based on studies conducted on English orthography. In the present study, we aimed to explore the handwriting effects on word recognition in both Chinese and English followed by word recognition between normal and dyslexic readers.

Chinese dyslexia differ from typical dyslexia in its written orthography, which is different from alphabetic languages, like English. The difference between visual processing of written orthography in Chinese and alphabetic languages has also been exacerbated by the fact that handwriting with Chinese characters differs from alphabetic writing such as that used in English. When handwriting Chinese, the visual-spatial features are extracted first, and then followed by visual-semantic mappings (Guan et al., 2011). In contrast, when an individual writes alphabetic words, phonological processing (i.e., mapping letters to phonemes), appears to be more crucial (Wagner et al., 1997; Ehri, 2014). Learning to read cannot be separated from handwriting in literacy development (James and Engelhardt, 2012; Tan et al., 2013; Ehir and Flugman, 2018). Handwriting practice and instruction are also essential to children's writing skills (Daly et al., 2003; van Reybroeck and Michiels, 2018) and reading development in Chinese (Guan et al., 2011, 2021; Tan et al., 2013) and western languages (James, 2010). Nevertheless, there is a dearth of research in handwriting effects of multisensory integration as a pathway to neural specialization for print in terms of word recognition among typical and dyslexic readers across writing systems.

Handwriting influences symbol learning by activating a neural network incorporating both motor and sensory routines in the human brain (Dehaene and Cohen, 2011). The motor system produces variability (*via* handwriting in this case) that promotes behavioral performance and connects brain systems to functional networks (James, 2017). Moreover, much research with both Chinese beginning readers and native English-speaking adults has demonstrated that handwriting Chinese characters highlights strokes, the basic constituents of the orthographic representation of the Chinese characters, and therefore enhances orthographic recognition, facilitating Chinese learners' reading acquisition (Longcamp et al., 2006; James, 2010; Guan et al., 2011, 2015, 2021). Interestingly, drawing squared shapes or line drawing also seem to enhance cognitive ability in character acquisition among Chinese school-aged children (Tan et al., 2013). We can conclude from these studies that handwriting practice or some stroke-like drawing practices might be an important means to promote students' learning of written and spoken language.

There are some important theoretical merits for investigating the different recognition mechanisms associated with handwriting either curved or straight-line units, considering different orthographic features in Chinese and English. Above all, there are 26 letters in the English alphabet, some of which consist of curved lines (like “O, Q”), and others of which consist of straight lines (like “L, H”). English words consist of letter strings in a sequential order. The word recognition process takes place *via* an interactive scope including single letters at the local level and sight words at the global level, depending on the individual differences in words (including length and frequency) and readers (including language proficiency and familiarity with target words; Guan et al., 2020). Unlike English, Chinese orthography is composed of characters. Each character consists of 1–36 overlapping strokes. Strokes can be further arranged into logographemes and then radicals, some of which can also be stand-alone characters, but most of which are within-character subunits (Yu and Reichle, 2017). These subunits of writing consist of either curved or straight-line features (e.g., “心” in a curved shape or “王” in a straight-line shape), but the whole character occupies a uniformly-sized, two-dimensional square-shaped spatial layout in text. Therefore, the cognitive processes involved in word recognition induced by curved and straight-line handwriting might manifest differently in Chinese than in English. Meanwhile, cursive handwriting is a complex cultural skill (Kersey and James, 2013; Kiefer et al., 2015) that involves many brain systems and the integration of both motor and perceptual skills (Vinci-Booher et al., 2016; Thibon et al., 2019). Writing in a cursive manner is commonly used as a tool for acquiring handwriting skills (Arnold et al., 2017; Ose Askvik et al., 2020). Furthermore, handwriting of strokes helps Chinese learners improve orthographic recognition and orthographic-semantic mapping at both the character and lexical levels (Lyu et al., 2021), but the effect of handwriting single letters in English on word recognition remains unexplored. Thus, handwriting curved and straight-line writing units in either language might affect Chinese and English word recognition differently.

The N170 is an event-related potential (ERP) functioning as a neurophysiological indicator of early visual word recognition. The typography of N170 ERP responses demonstrates visual specialization for reading development (Maurer et al., 2005). Moreover, the N170 might indicate a orthographic processing strategy in visual word recognition, which involves selectivity and modulation of the brain regions (e.g., laterality or delayed latency) relating to recognizing the word form (Simon et al., 2007). In terms of expertise in reading Chinese (Zhao et al., 2012) and Japanese (Maurer et al., 2008), the laterization of the N170 serves as an electrophysiological marker as well. Nevertheless, it is still unknown whether handwriting experience modulates the N170. Although there are other early ERP indicators of visual processing (e.g., P1, N1), they are non-linguistic (Planton et al., 2013; Rothe et al., 2015) and are therefore not examined in the present study. Taken together, based on the previously described studies, it is innovative to examine the N170 modulation and its laterality effect involved with the different operationalization of handwriting and drawing practices in comparison to viewing. Furthermore, in the current study, we also considered the effect of curved vs. straight-line inputs as this feature might be crucial for visual-perceptual categorization in visual recognition (Seyll and Content, 2020). Hence, it is of great interest to reveal the effect on N170 modulation of handwriting followed by Chinese recognition in comparison to drawing followed either by Chinese or English recognition.

In summary, there is theoretical merit to explore to what extent handwriting symbols in comparison to drawing or viewing word-like symbols promotes the perception of word recognition in both Chinese and English. Whether handwriting Chinese might promote early visual perception more than drawing shapes or the control condition of viewing characters is still unexplored. Moreover, it is worthy of further examining the handwriting or drawing effects between typical readers and their counterparts who might have disabilities in reading development.

## The Current Study

We investigate both condition and stimuli effects between typically developing and dyslexic readers. First, the current study focuses on the condition effect, i.e., the difference between handwriting Chinese (HC), and viewing Chinese (VC) as a control; and the difference between drawing followed by Chinese recognition (DC) and drawing followed by English recognition (DE). Second, the current study also focuses on the stimuli effect of either curved or straight lines. Specifically, we focus on the early visual ERPs indicator of N170, and aims to explore the effect of four learning conditions on the underlying different neural mechanism word recognition. The following research questions guide the present investigation:

1. Whether and to what extent does the handwriting effect exist in word recognition in typical and dyslexic readers in terms of behavioral and ERP responses;
2. Whether and to what extent does the drawing effect exist in word recognition in typical and dyslexic readers in terms of behavioral and ERP responses;
3. Whether and to what extent does the stimuli effect (curved line vs. straight line strokes) exist in Chinese character recognition in handwriting in terms of behavioral and ERP responses;
4. Whether and to what extent does the stimuli effect (curved vs. straight-line shape) exist in English word recognition in drawing in terms of behavioral and ERP responses;
5. How different is it in the laterality effect of the handwriting and drawing on word recognition across languages (e.g., Chinese character vs. English word recognition) between typical and dyslexic readers?

## Method

### Participants

The University of Science and Technology Beijing (USTB) ethics committee approved the study. The consent forms were signed first, and a background language experience survey were also completed by individual parents of the participants. The survey also included questions about children's developmental disorders and learning disabilities. After screening, 21 children (15 males, *M*_age_ = 9.5 years, SD = 0.86) in grades three and four, in which handwriting instruction has just been introduced and is thus considered as the critical period of handwriting development, participated in the experiment. Dyslexic readers were also diagnosed from a pool of nearly 450 grade 4, 5, and 6 students from elementary school. After screening, 21 children (17 males, M_age_ = 9.2 years, SD_age_ = 0.86) in grades three and four participated in the experiment.

To be diagnosed as having dyslexia, children's checklist composite score and at least three sets of cognitive-linguistic composite performance needed to be at least 1 SD below the means of their respective age groups on the parent-report scale of Dyslexia Checklist for Chinese Children (DCCC; Hou et al., 2018), which included ten constructs based on 57 items, i.e., vocabulary, visual word recognition, auditory word recognition, spelling, written expression, attention, oral language, and bad reading habits, as well as family risk of dyslexia and mathematic ability. The theoretical framework of this checklist is based on the standard definition of developmental dyslexia in ICD-10, DSM-IV, and clinical symptoms defined by Liu and her colleagues (Liu and Perfetti, 2003). The DCCC is a standard and well-established rating scale for Chinese dyslexia with good reliability and validity. Higher DCCC scores correspond to lower reading ability. In the current measure, the difference in reading-related scores based on the DCCC were statistically significant between dyslexic and normal children in our sample (*t* < 0.05). All the participants were right-handed, with normal or corrected-to-normal vision, and no history of neurological disorders based on screening tests. The intelligence quotients of our selected participants were all above 80, as assessed by Wechsler Intelligence Scale for Children (WISC)-IV Chinese Version. There was no variability in participants' English proficiency as they were English beginners. At the point of the experiments, they had been taught all 26 letters at school and were familiar with all of the stimuli. We offered the stipends for both traveling to the from the experimental sites and their accommodation fees. Each individual participants were also paid with 80 yuan (approximately $11 USD) per hour.

### Materials

We selected both Chinese characters and English words from children's curriculum details about the selection process can be found in Guan et al. (2020, 2021) and Guan and Fraundorf (2020). The materials included the prompt, target 1, and target 2. Chinese prompt stimuli included six-curved-line characters (心, 乙, 人, 飞, 九, 儿), and six straight-line characters (口, 工, 日, 王, 十, and 田). The total of 32 target1-characters were selected according to the following three criteria: (1) high frequency (Chen and Shu, 2001); (2) easy to be embedded within in target-2 characters; and (3) contained either curved- or straight-line strokes. Target 2 comprised compound characters that contained the target 1 characters, so the compound target 2 must have more strokes than that of target 1. The curve and straight features of the prompts and the targets were counterbalanced. The sample stimuli are shown in Supplementary Appendix 1.

The English materials consist of capital letters and words. The stimuli-to-be learned in the learning condition were six straight-line letters and four curved letters (like H, F, I, T, E, L, O, C, Q, and U). Thirty-two target 1 contained all 26 capital letters. The word length of the 32 target 2 words contained no more than 6 letters in caps. Before training, we made sure that all participants were familiar with the forms of these words. Therefore, the words chosen were known by all participants, which controlled for the effect of familiarity. Because participants were familiar with all of the stimuli, learning should not be affected by priming as all four conditions (three experimental conditions and one control condition of viewing) shared experimental stimuli with similar features and the only differences lied in the learning procedure. Even if a priming effect were present, comparison between conditions should cancel it out. The judgment task was the same for both Chinese and English: to decide whether target 1 was embedded in target 2. The sample stimuli are shown in Supplementary Appendix 2.

**Figure 1:**
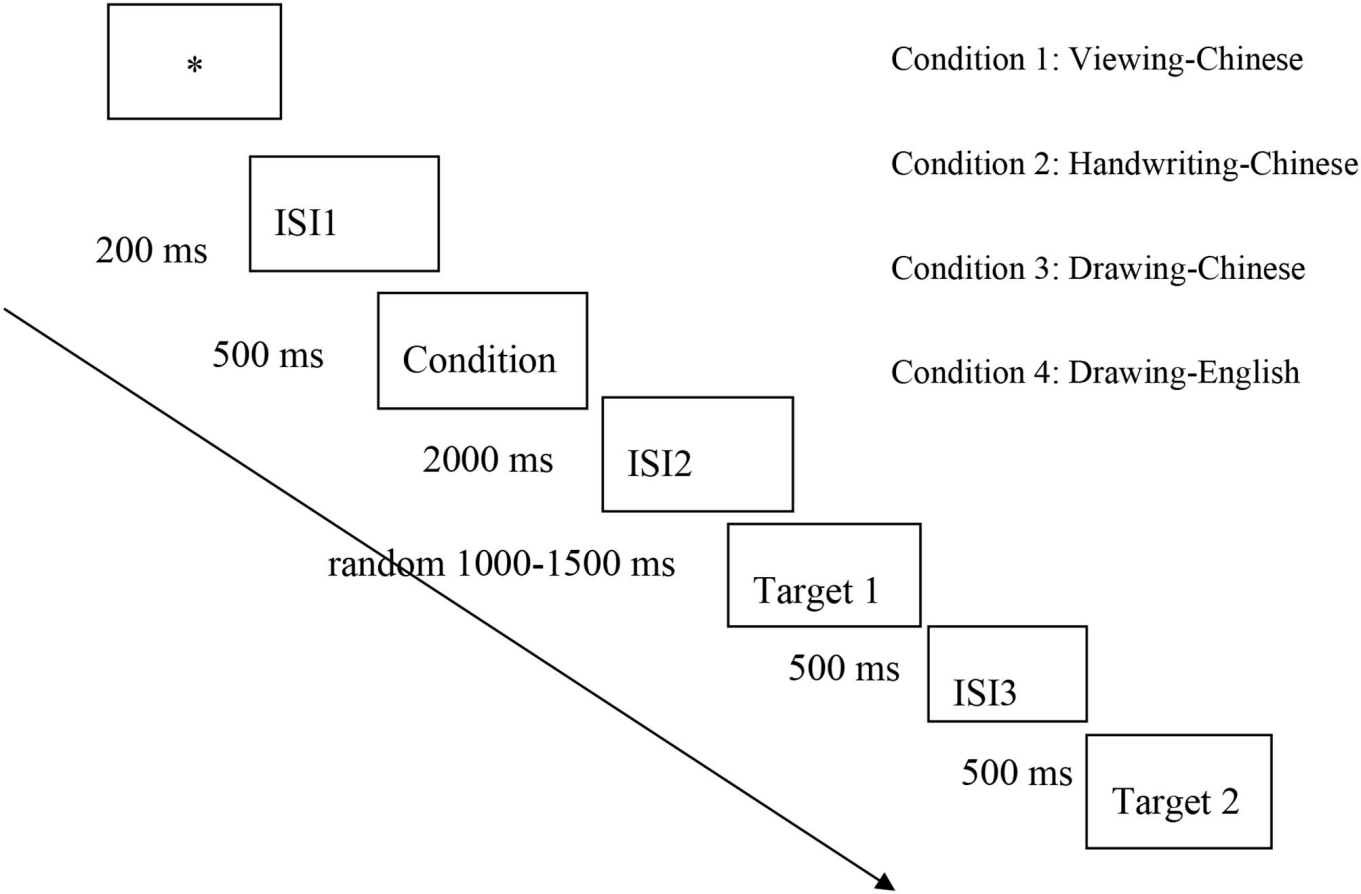
Experimental procedure.

For two drawing condition, the stimuli containing 4 curved-line drawing images (circle, heart, moon, and approximate equal), and 4 straight-line drawing images (rectangle, cross, rising line, and horizontal line). Pleases refer to Supplementary Appendices 1, 2 for details. After drawing the images, the participants were required to make the yes or no judgement task on whether target 1 was embedded in target 2 (embedment judgement task). In the drawing condition, we also designed an equal number of control trials in which no visual image of the prompt is shown before the embedded judgement task. To compare the curved vs. straight stimuli effects on word recognition, blank trials were used as a control. Please see Figure 1 design flow chart for the procedure of the stimuli presentation.

### Procedures

A within-subject design was carried out. Four conditions were treated as independent variable; behavioral performance (accuracy and response time) and the magnitudes of N170 ERP component were treated as the dependent variables for different research questions.

There were four learning conditions. The first learning condition was viewing-Chinese (VC), under which participants viewed Chinese word stimuli and then responded to the judgment target task by making a binary decision on whether target 2 contained target 1. The second condition was handwriting-Chinese (HC), in which participants wrote simple Chinese character stimuli on a writing pad and then responded to the same Chinese judgment target task. The drawing condition followed by Chinese recognition (DC) required the participants to draw the prompt (circle, square, triangle, diamond, rectangle, parallel lines, or wavy lines) on the writing pad first and then respond to the embedment judgment task in Chinese. The drawing followed by English recognition (DE) asked the participants to draw the same prompts as those in the DC condition and the responded to the judgement task in English. The order of the four conditions in this experiment was counterbalanced.

Each participant participated in an electroencephalogram (EEG) test with a total duration of 350 s. The data was collected in the EEG laboratory of the National Institute of Education Science, and all materials appeared in the center of the computer screen. Before the formal experiment, participants participated in a training activity designed to familiarize them with the experimental procedures in all four conditions. See Figure 1 for the flowchart of the presentation. To start, a fixation asterisk appeared on the screen for 200 ms; following the fixation, a blank black screen appeared for 300 ms. Then there was a 2,000 ms learning phase. In all four conditions, the learning phase began with the stimulus in blue, followed by target 1 in red, and then target 2 in white. In the handwriting condition, participants wrote the blue stimulus. In the viewing condition, participants spent the same length of time viewing the stimuli. After a blank black screen appeared for 1,000–1,500 ms (duration chosen at random), the red target 1 was shown to participants for 500 ms followed by a 500-ms blank black screen. Finally, target 2 in appeared in white, and participants was instructed to press button “y” if target 2 included target 1 or button “n” if it did not. In a word, participants decided whether target 1 was included in target 2. When participants pressed the button, the stimulus disappeared; if no button was pressed, the stimulus remained for 3,500 ms. The program then advanced to the next trial. EEG recording began upon the onset of the fixation and proceeded continuously, during which responses to target 1 and target 2 were all marked in the EEG recording.

### ERP Data Acquisition and Pre-processing

Response time and accuracy were recorded during EEG data acquisition. EEG data was collected using NeuroScan's ESI-64 system. Electrode position in this study approximated locations of the international 10–20 system. The study used the left mastoid as the reference electrode. The vertical electrooculogram (VEOG) was recorded by using two electrodes placed above and below the midline of the right eye, and the recording electrodes of the horizontal electrooculogram (HEOG) were placed beside the left and right eyes in horizontal alignment with the eyeball.

All electrodes were placed on the scalp using conductive paste to ensure that the impedance of each electrode was kept below 5 KΩ. The EEG data acquisition software was NEUROSCAN. The amplifier was SYNAMPS2, and AC continuous sampling was adopted. Scalp potentials were recorded with a sampling rate of 1,000 Hz, and the bandpass filter is 0.05~100 Hz.

Offline analysis of EEG data was performed using Curry 7.0. During the recording, the left mastoid was used; later, the data was referenced offline using a reference averaged across left and right mastoids. First, a constant baseline correction was performed. Second, the data was digitally filtered with a 30-Hz lowpass. Then, the components related to eye movement were removed. In addition, amplitudes exceeding ±100 μV were also excluded as artifacts. The continuous EEG data was segmented, with the duration of the segmentation starting 200 ms before the onset of target 1 and extending 800 ms after target 1. Finally, the ERP components were superimposed and averaged, and the baseline correction was performed using the baseline of 200 ms before the stimulus.

### Behavior and ERP Data Analyses

For behavioral data, we conducted 4 (learning conditions: VC, HC, DC, and DE) × 2 (normal vs. dyslexic readers as between-subject factor) repeated measures ANOVAs on response time and accuracy.

For ERP data, according to prior literature (Maurer et al., 2008), the N170 component elicited by Chinese characters has generally been recorded *via* PO7 and PO8 electrodes, and a lateralization effect has been reported, with the left negative wave larger than the right negative wave (Rossion et al., 2007; Zhang et al., 2011). The stimulus-elicited peak and latency of the N170 at the PO7 and PO8 electrodes of each participant were extracted from the ERP data and analyzed *via* statistical models using SPSS 17.0.4. Four (learning conditions: VC, HC, DC, and DE) × 2 (electrode position: left PO7 and right PO8) repeated measures analyses of variance (ANOVAs) were performed to analyze the amplitude and latency of the N170 of both normal and dyslexic readers. After demonstrating a significant main effect of group and learning condition, as well as their interaction, we broke the analyses down into two groups (normal and dyslexic readers). To answer the first two research questions, we compared three pairs of learning conditions (VC vs. HC, HC vs. D-C, HC vs. DE) in the normal and dyslexic readers groups respectively.

To answer the third and fourth research questions regarding stimuli and laterality effects, we conducted Stimuli (curved vs. straight-line) × Laterality (PO7 vs. PO8) analyses on both behavioral data and hemispheric differences in the N170. A Bonferroni correction was used to correct for multiple comparison as the data violated the assumption of sphericity (Blan and Altman, 1995; Chen et al., 2017). We used 0.05 significance level for all analyses.

## Results

### Behavioral Results

Differences in the behavioral analyse between the two groups of normal and dyslexic readers could be only related to their cognitive ability (Palmis et al., 2020), as we used the same materials, same training procedures. We did not focus on comparisons between normal and dyslexic readers directly. Instead we investigated the differences in behavioral results in the pairs of four learning conditions between the normal and dyslexic readers.

**Figure 2:**
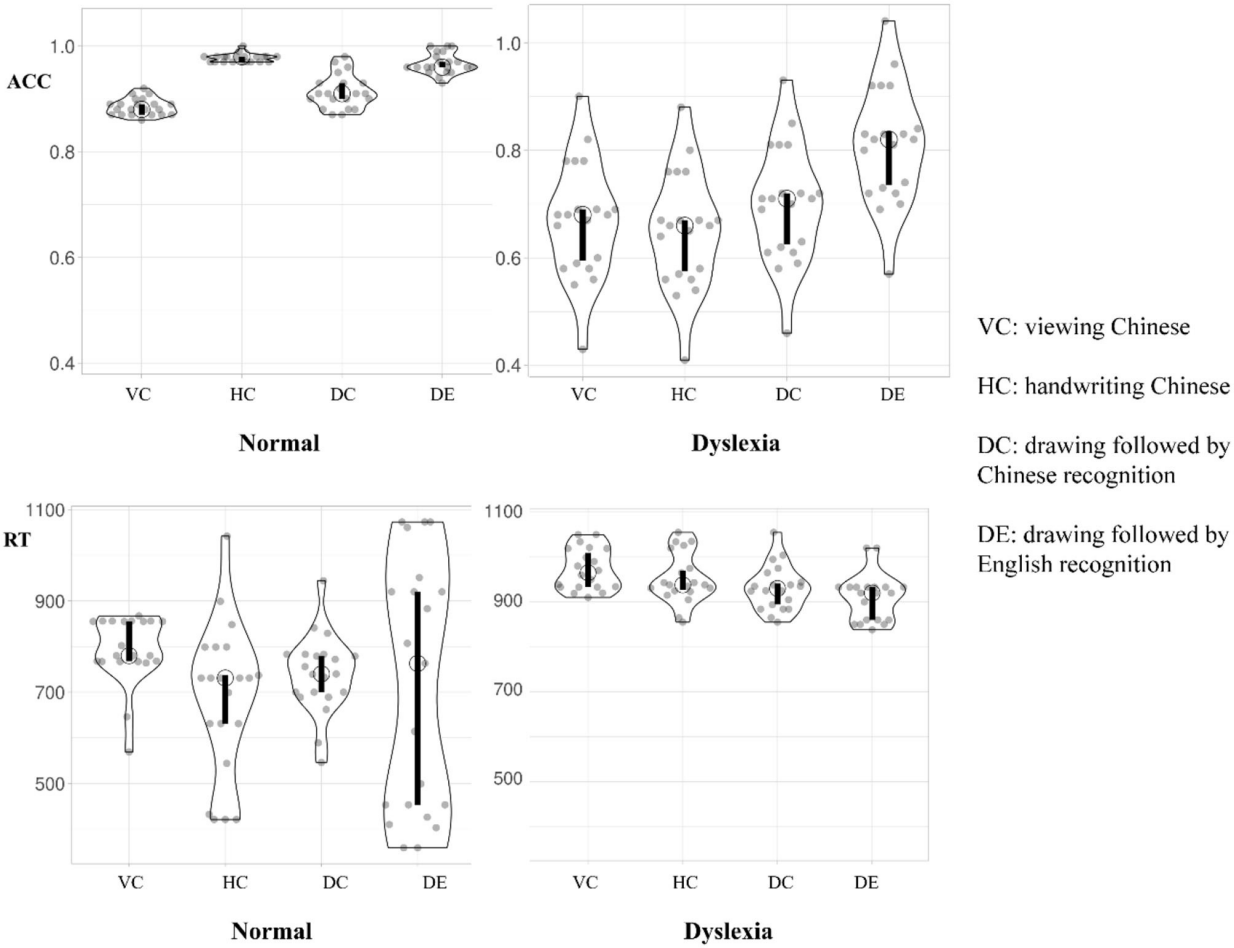
Behavioral data of normal readers and dyslexic children. Open circle indicates the median in each condition. Bar chart indicates the 95% confidence interval for each median determined by bootstrapping.

For behavioral data analyses, we collected both accuracy (ACC) and response time (RT) for target 2. Accuracy analyses were based on the aggregated means per subject per condition. We recorded the response time (RTs) at the onset of target 2 button press. The analyses also excluded the outliers in RTs in the extreme 5% on either end of the Z-normalized distribution of RTs (i.e., above and below 1.65 SD of each mean RT per participant). At last, 7.5% of trials being excluded as outliers, following the criteria (from 5 to 10%) suggested by Ratcliff (1993). The descriptive statistics of mean and standard deviation of both ACC and RT for each of four conditions per groups are shown in Table 1. The violin plots summarizing the behavioral data for both normal readers and dyslexic readers are present in Figure 2.

**Table 1:** Mean and SD of both ACC and RTs in the four conditions.

**Condition**	**RT**			**ACC**		
	**Normal**	**Dyslexic**	**Cohen's** ***d***	**Normal**	**Dyslexic**	**Cohen's** ***d***
VC	1,795.05 (76.90)	1,973.15 (46.80)	1.71	0.89 (0.02)	0.67 (0.11)	1.44
HC	1,688.90 (70.20)	1,952.85 (55.66)	1.60	0.98 (0.01)	0.65 (0.11)	3.28
DC	1,742.35 (86.90)	1,931.45 (49.80)	1.63	0.91 (0.03)	0.70 (0.11)	0.36
DE	1,725.70 (70.10)	1,909.95 (52.21)	1.51	0.87 (0.02)	0.83 (0.11)	1.17

*RT, response time; VC, viewing character; HC, handwriting character; DC, drawing followed by Chinese recognition; DE, drawing followed by English recognition. Standard deviation of each measure per condition presented in parentheses. We calculated Cohen's d by using the following formula: [4η^2^/1-*η*^2^]^1/2^. Cohen's d < 0.2 indicates a small effect size, 0.2 < Cohen's d < 0.8 indicates a medium effect size, and Cohen's d > 0.8 indicates a large effect size (Fritz et al., 2012)*.

Four repeated-measures analyses of variance (ANOVAs) were performed using a single factor (learning conditions: VC, HC, DC and DE), by submitting response time and accuracy for each condition across normal and dyslexic readers. The group (normal vs. dyslexic) factor was used as the between-participant factor. Response time and accuracy of normal and dyslexic readers demonstrated significant effects of learning condition. For response time, there was a significant effect of learning condition [*F*_(3, 57)_ = 24.71, *p* = 0.04, *η*^2^ = 0.029] and condition × group interaction [*F*_(3, 57)_ = 10.03, *p* = 0.04, *η*^2^ = 0.01]; for accuracy, there was a significant effect of learning condition [*F*_(3, 57)_ = 861.88, *p* < 0.01, *η*^2^ = 0.09] and a significant condition × group interaction [*F*_(3, 57)_ = 470.49, *p* < 0.01, *η*^2^ = 0.05]. Therefore, three sets of *post-hoc* analyses were carried out below in normal and dyslexic readers, respectively.

### Handwriting Effects in Comparison to Other Learning Conditions in Normal vs. Dyslexic Readers

#### Comparing Handwriting vs. Viewing

Among normal readers, the response time in HC (*M* = 1,688.90 ms, *SD* = 70.26) was significantly shorter than VC (1,795.05 ms, *SD* = 76.95), [*F*_(1, 38)_ = 6.46, *p* = 0.02, *η*^2^ = 0.15], and the accuracy rate in HC (*M* = 0.98, *SD* = 0.01) was significantly higher than in VC (*M* = 0.89, *SD* = 0.02), [*F*_(1, 38)_ = 512.97, *p* < 0.01, *η*^2^ = 0.93]. For dyslexic readers, the patterns were the same. Their response time in HC (*M* = 1,952.85 ms, *SD* = 55.66) was shorter than VC (*M* = 1,973.15 ms, *SD* = 46.80), [*F*_(1, 38)_ =1.568, *p* = 0.218, *η*^2^ = 0.04], and accuracy of HC (*M* = 0.65, *SD* = 0.11) was lower than VC (*M* = 0.67, *SD*=0.11), [*F*_(1, 38)_ = 0.32, *p* = 0.574, *η*^2^ =0.01].

#### Comparing Handwriting vs. Drawing Followed by Chinese Recognition

For normal readers, the response time in HC (*M* = 1,688.90 ms, *SD* = 170.26) was shorter than DC (*M* = 1742.35 ms, *SD* = 86.90), [*F*_(1, 38)_ = 1.56, *p* = 0.22, *η*^2^ = 0.04], and the accuracy in HC (*M* = 0.98, *SD* = 0.01) was significantly higher than DC (*M* = 0.91, *SD* = 0.03), [*F*_(1, 38)_ = 72.27, *p* < 0.001, *η*^2^ = 0.66]. For dyslexic readers, there was a longer response time of HC (*M* = 1,952.85 ms, *SD* = 55.66) compared with DC (*M* = 1,831.45 ms, *SD*=49.80), [*F*_(1, 38)_ = 1.64, *p* = 0.21, *η*^2^ = 0.04] and accuracy in HC (*M* = 0.65, *SD* = 0.11) was lower than DC (*M* = 0.70, *SD* = 0.11), [*F*_(1, 38)_ = 2.07, *p* = 0.16, *η*^2^ = 0.05].

#### Comparing Drawing Followed by Chinese Recognition vs. Drawing Followed by English Recognition

For normal readers, the response time of Chinese recognition in the DC condition (*M* = 1,742.35 ms, *SD* = 86.90) was not significantly different from English recognition in the DE condition (*M* = 1,725.70, *SD* = 270.10), [*F*_(1, 38)_ = 0.069, *p* = 0.79, *η*^2^ = 0.002], but the accuracy of DC (*M* = 0.91, *SD* = 0.03) was significantly lower than the DE condition (*M* = 0.97, *SD* = 0.03), [*F*_(1, 38)_ = 39.97, *p* < 0.001, *η*^2^ = 0.51]. This pattern of results might not be affected by the condition effect between DC and DE, but by the fact that the normal readers felt more familiar with the English stimuli than the Chinese stimuli. For dyslexic readers, there was no difference in response time (*p* = 0.28), and no significant difference between accuracy with DE higher than DC either (*p* = 0.17).

Based on the above analysis, the results suggest that there is a significant handwriting effect among normal readers and a significant drawing effect. Thus, we further analyzed the stimuli effect of curved- and straight-line characters in handwriting and drawing.

#### Comparing Curved-Line vs. Straight-Line Handwriting in Chinese

For normal readers, the response time for curved-line characters (*M* = 1,700.19 ms, *SD* = 172.12) was higher than for straight-line characters (*M* = 1,672.10 ms, *SD* = 154.04), [*F*_(1, 38)_ = 0.28, *p* = 0.60, *η*^2^ = 0.007], and the accuracy for curved-line characters (*M* = 0.96, *SE* = 0.01) was lower than straight-line characters (*M* = 0.97, *SE* = 0.02) [*F*_(1, 38)_ = 0.714, *p* = 0.403, *η*^2^ = 0.02].

#### Comparing Curved-Line vs. Straight-Line Drawing Followed by English Recognition

For dyslexic readers, there was a longer response time for curved-line drawing (*M* = 1,918.55 ms, *SD* = 47.31) compared with straight-line drawing (*M* = 1,801.35 ms, *SD* = 61.69), [*F*_(1, 38)_ = 0.93, *p* = 0.34, *η*^2^ = 0.02] and accuracy for curved line drawing (*M* = 0.85, *SD* = 0.09) was significantly higher than straight-line drawing (*M* = 0.76, *SD* = 0.12), [*F*_(1, 38)_ = 6.2, *p* = 0.013, *η*^2^ = 0.15].

### ERP Results

**Figure 3:**
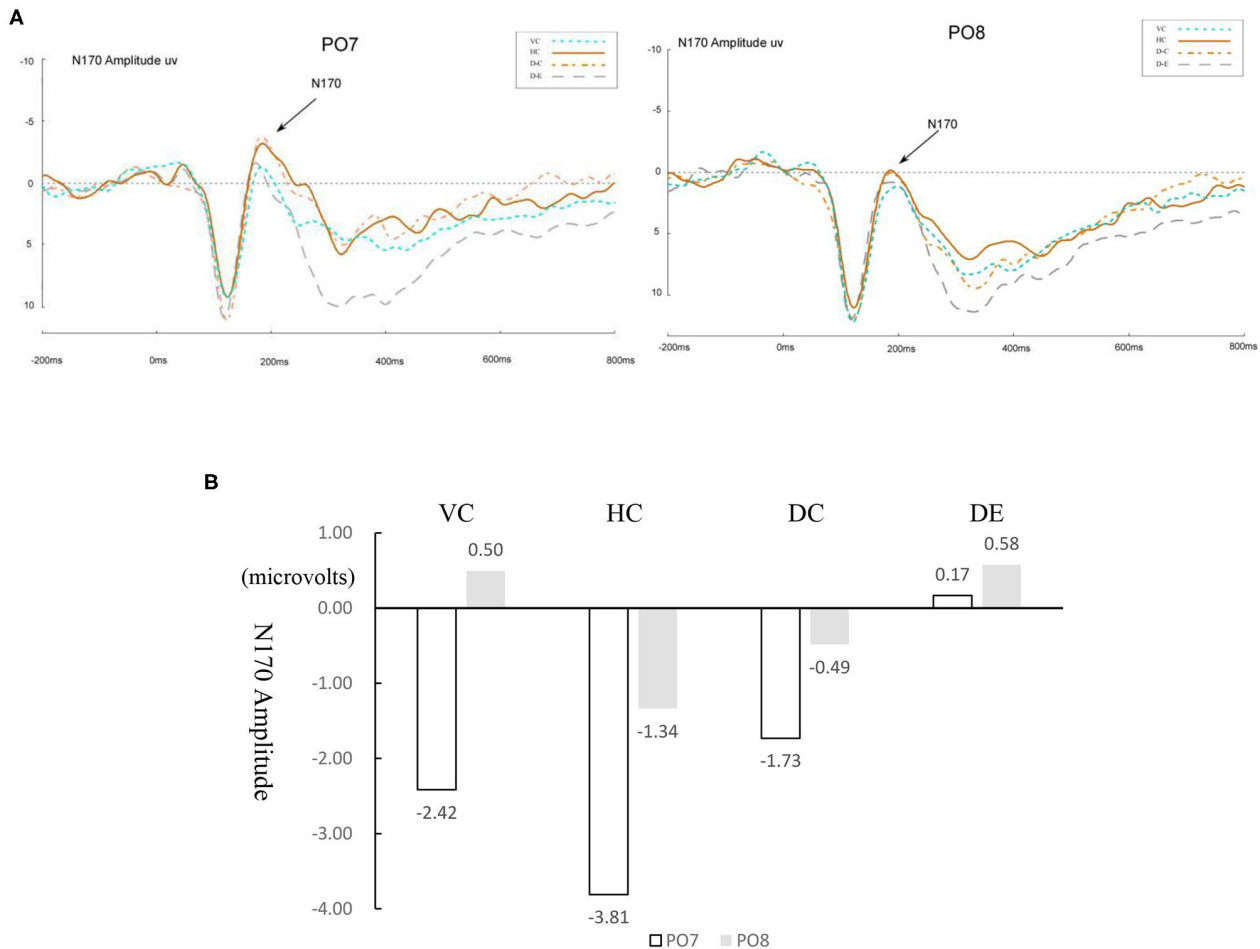
**(A)** ERP waveforms of the N170 under four conditions for normal readers for the left (PO7) and right (PO8) parietal leads. VC, viewing-Chinese; HC, handwriting-Chinese; DC, drawing followed by Chinese recognition; DE, drawing followed by English recognition. **(B)** Differences between four conditions for normal readers in the amplitude of N170.

**Figure 4:**
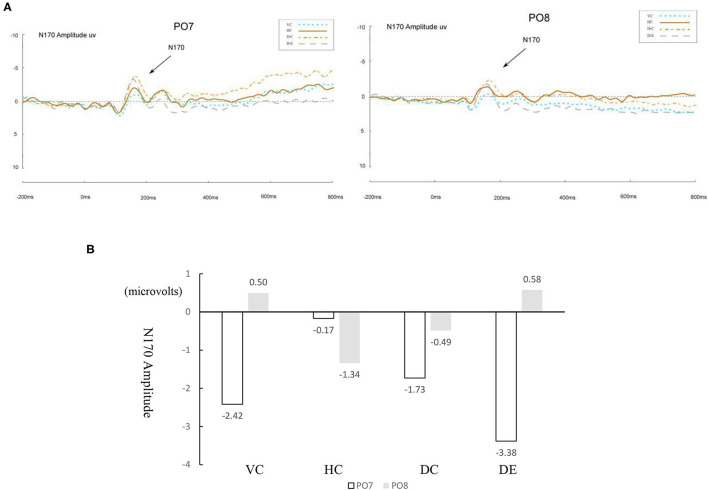
**(A)** ERP waveforms of N170 under four conditions for dyslexic readers for the left (PO7) and right (PO8) parietal leads. VC, viewing-Chinese; HC, handwriting-Chinese; DC, drawing followed by Chinese recognition; DE, drawing followed by English recognition. **(B)** Differences between four conditions for dyslexic readers in the amplitude of N170.

Figures 3A,B presents the waveforms of ERP modulations that marked target 2 responses at PO7 and PO8 for normal readers, and Figures 4A,B for dyslexic readers. A 4 (learning conditions) × 2 (hemisphere: left PO7 and right PO8) × 2 (group: normal vs. dyslexic) × 2 (stimuli: curved vs. straight) repeated measures ANOVA was conducted on the amplitude of N170. The results revealed significant main effects of condition [*F*_(3, 60)_ = 4.72, *p* = 0.005, *η*^2^ = 0.02] and hemisphere [*F*_(1, 20)_ = 18.98, *p* < 0.001, *η*^2^ = 0.076], and a significant condition × hemisphere × group interaction [*F*_(3, 60)_ = 11.02, *p* < 0.001, *η*^2^ = 0.04]. Moreover, we found a significant condition × hemisphere 2-way interaction [*F*_(3, 60)_ = 7.07, *p* < 0.001, *η*^2^ = 0.019], and significant group × condition two-way interaction [*F*_(3, 60)_ = 10.21, *p* < 0.001, *η*^2^ = 0.04]. This indicates a different pattern across hemispheres between conditions and between the two groups, and also a significant group × hemisphere × stimuli three-way interaction [*F*_(2, 40)_ = 9.34, *p* < 0.001, *η*^2^ = 0.032].

Therefore, the ERP analyses on the N170 amplitude were conducted to test comparison between normal and dyslexic groups separately in each of all four conditions. The descriptive statistics of ERP data are shown in Table 2. We only reported the amplitude data. Previous studies (Maurer et al., 2008; Yum et al., 2014; Yum and Law, 2021) did not find the statistical significance on the latency. Figures 3B, 4B show the differences in amplitude voltage between the conditions for normal and dyslexic separately.

**Table 2:** Mean (SD) ERP magnitude at PO7 and PO8 for four conditions.

	**Normal Readers**		**Dyslexic Readers**	
	**PO7**	**PO8**	**PO7**	**PO8**
VC	−2.42 (3.98)	0.50 (2.67)	−2.42 (3.98)	0.50 (2.67)
HC	−3.81 (3.22)	−1.34 (3.10)	−0.17 (2.77)	−1.34 (3.10)
DC	−1.73 (2.88)	−0.49 (3.34)	−1.73 (2.88)	−0.49 (3.34)
DE	0.17 (2.68)	0.58 (2.83)	−3.38 (3.84)	0.58 (2.83)

*VC, viewing character; HC, handwriting character; D-C, drawing followed by Chinese recognition; D-E, drawing followed by English recognition. Standard deviation of each measure per condition is presented in parentheses*.

**Figure 5:**
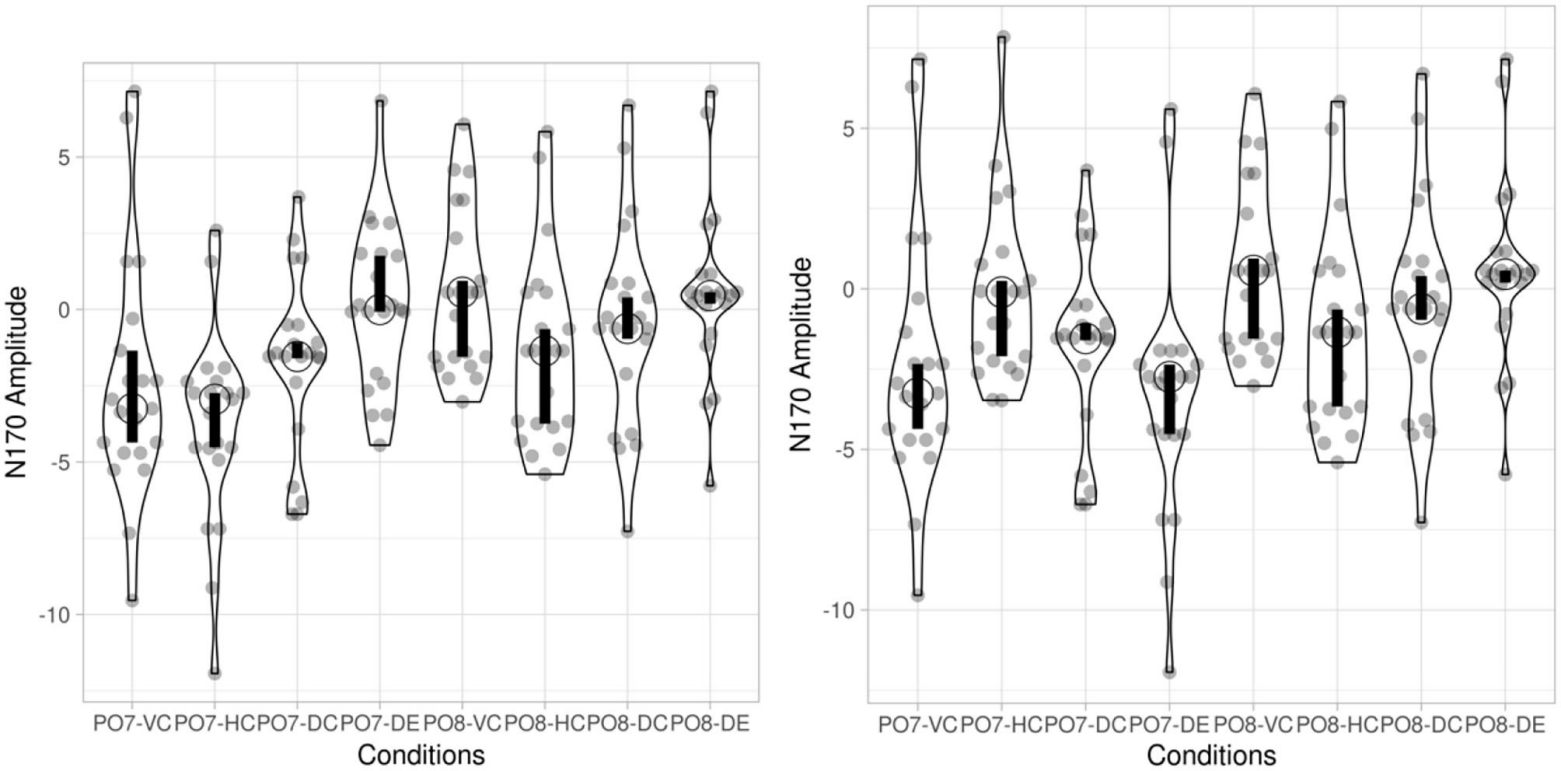
N170 amplitude of normal readers (left) and dyslexic readers (right) in VC, HC, DC and DE condition. Open circle indicates the median of the data. Bar chart indicates the 95% confidence interval for each median determined by bootstrapping. (VC, viewing-Chinese; HC, handwriting-Chinese; DC, drawing followed by Chinese recognition; DE, drawing followed by English recognition).

**Figure 6:**
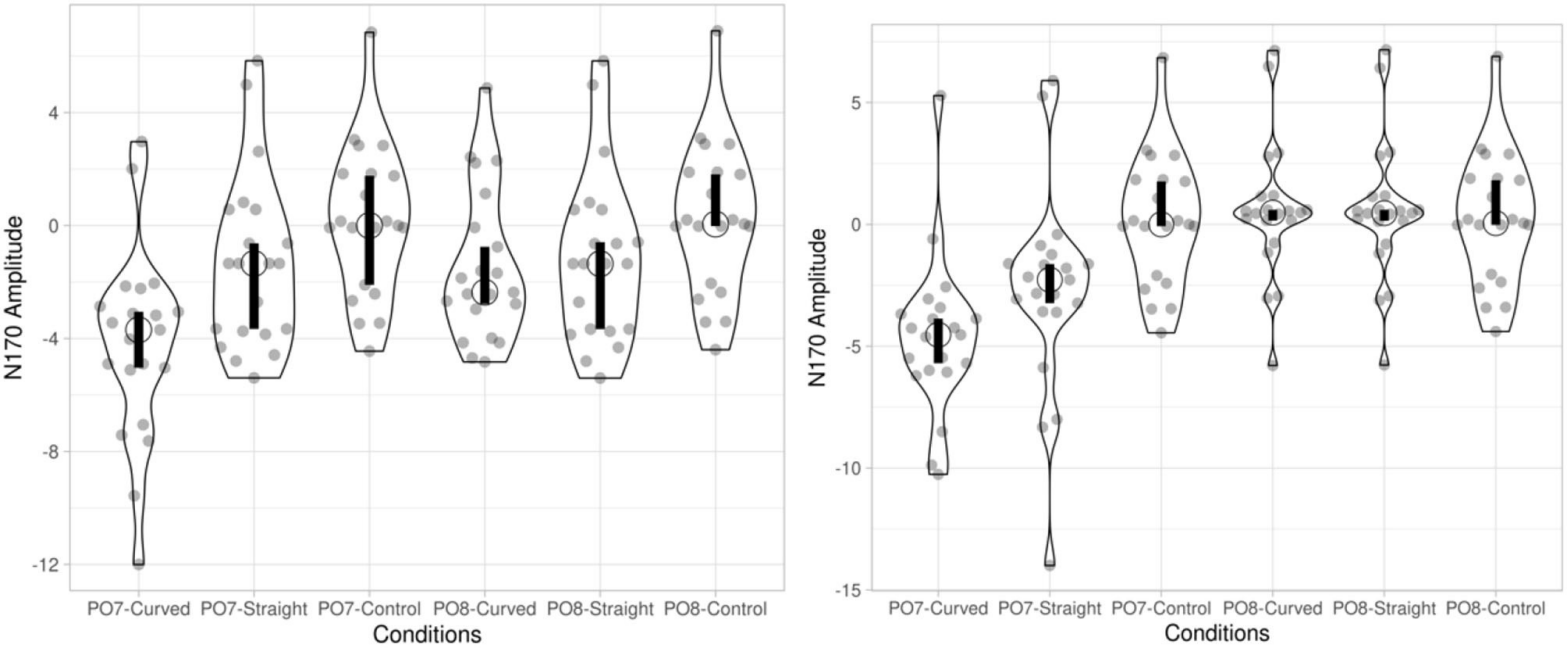
N170 amplitude of normal readers (left) and dyslexic readers (right) in straight, curved and control condition. Open circle indicates the median of the data. Bar chart indicates the 95% confidence interval for each median determined by bootstrapping. (Left: curved: handwriting curved; straight: handwriting straight, control: handwriting control. Right: curved: drawing English curved; straight: drawing English straight; control: drawing English control).

Figures 5, 6 present violin plots summarizing the ERP amplitude voltage data for both normal and dyslexic readers.

**Figure 7:**
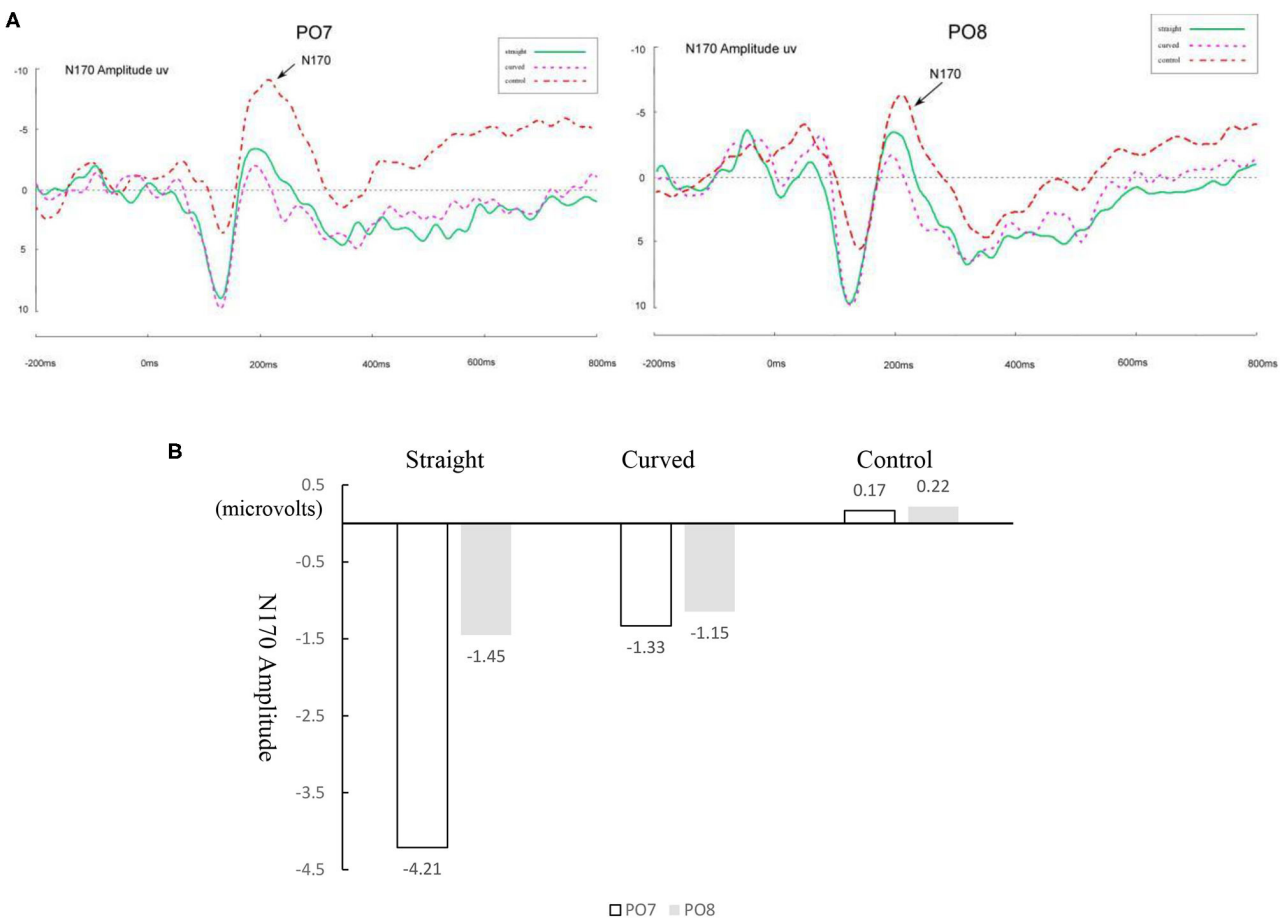
**(A)** ERP waveforms of N170 under straight, curved control handwriting condition for normal readers for the left (PO7) and right (PO8) parietal leads. **(B)** Differences between three conditions for normal readers in the amplitude of N170.

**Figure 8:**
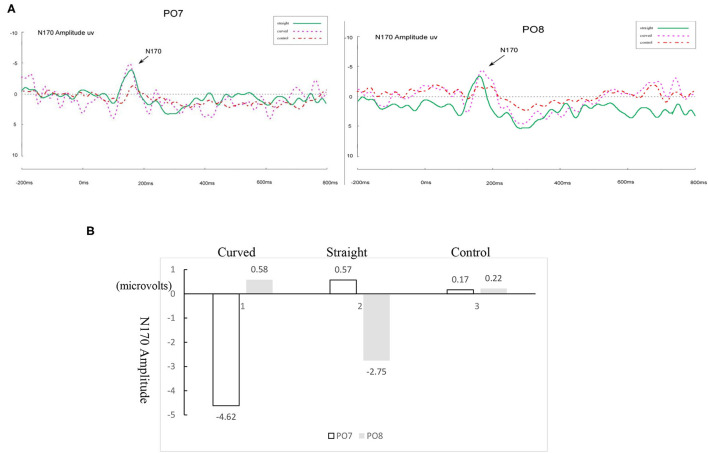
**(A)** ERP waveforms of N170 under straight, curved control drawing English condition for dyslexic readers for the left (PO7) and right (PO8) parietal leads. **(B)** Differences between three conditions for dyslexic readers in the amplitude of N170.

To better show the handwriting effect, Figure 7A presents the original ERP waveforms modulated by stimuli (curved, straight and control) with target 1 responses at PO7 and PO8 marked for normal readers, and Figure 7B is the voltage comparison. To better show the drawing effect, Figure 8A presents the original ERP waveforms modulated by stimuli (curved, straight and control) with target 1 responses at PO7 and PO8 marked for dyslexic readers, and Figure 8B is the voltage comparison.

#### Comparing Handwriting vs. Viewing

For normal readers, there was a greater N170 amplitude during HC than VC, *F*_(1, 15)_ = 0.72, *p* = 0.035, *η*^2^ = 0.03, showing that handwriting facilitates recognition of Chinese characters. For dyslexic readers, this pattern was the same. The amplitude of the N170 was significantly greater for HC than for VC, *F*_(1, 15)_ = 1.879, *p* = 0.03, *η*^2^ = 0.06.

#### Comparing Handwriting vs. Drawing Followed by Chinese Recognition

For both normal and dyslexic readers, there was no difference in N170 amplitude for HC vs. DC, [*F*_(1, 15)_ = 2.191*, p* > 0.05, *η*^2^ = 0.068 for normal readers; *F*_(1, 15)_ = 0.473*, p* > 0.05, *η*^2^ = 0.02 for dyslexic readers].

#### Comparing Drawing Followed by Chinese Recognition vs. Drawing Followed by English Recognition

For normal readers, DC elicited a significantly larger N170 response than DE, *F*_(1, 15)_ = 15.07, *p* = 0.02, *η*^2^ = 0.53. For dyslexic readers, N170 amplitude was also greater for DC than DE, *F*_(1, 15)_ = 0.527, *p* = 0.04, *η*^2^ = 0.02.

#### Laterality Effect

For normal readers, the peak value of N170 in the left hemisphere (PO7) was significantly higher than that in the right hemisphere (PO8) for HC [*F*_(1, 40)_ = 6.43, *p* = 0.015, *η*^2^ = 0. 138], VC [*F*_(1, 40)_ = 7.75, *p* = 0.008, *η*^2^ = 0.162], but laterality effects were not significant in the two drawing conditions [DC: *F*_(1, 40)_ = 1.68, *p* = 0.20, *η*^2^ = 0.04; DE: *F*_(1, 40)_ = 0.23, *p* = 0.64, *η*^2^ = 0.006].

For dyslexic readers, the peak value of N170 in the left hemisphere (PO7) was significantly higher than that in the right hemisphere (PO8) for VC [*F*_(1, 40)_ = 7.75, *p* = 0.008, *η*^2^ = 0.16] and for DE [*F*_(1, 14)_ =14.46, *p* < 0.001, *η*^2^ = 0.27]. The other two conditions showed no significant laterality effect [HC: *F*_(1, 40)_ = 1.64, *p* = 0.21, *η*^2^ = 0.04; DC: *F*_(1, 40)_ = 1.68, *p* = 0.20, *η*^2^ = 0.04].

#### Comparing Curved vs. Straight-Line Handwriting on Chinese Word Recognition

For normal readers, the peak value of N170 in the left hemisphere (PO7) was significantly higher than that in the right hemisphere (PO8) for straight-line handwriting [*F*_(1, 40)_ = 8.55, *p* = 0.006, *η*^2^ = 0.18]. Laterality effects were not significant for the curved line condition [*F*_(1, 40)_ = 0.04, *p* = 0.847, *η*^2^ = 0.09] or the control condition [*F*_(1, 40)_ = 0.004, *p* =0.95, *η*^2^ = 0.09].

#### Comparing Curved vs. Straight-Line Drawing on English Word Recognition

For dyslexic readers, the peak value of N170 in the left hemisphere (PO7) was significantly higher than that in the right hemisphere (PO8) for curved-line drawing followed by English recognition [*F*_(1, 40)_ = 30.79, *p* < 0.001, *η*^2^ =0.44], but laterality effects were not significant in the straight-line drawing condition and control condition (*p* < 0.01).

Table 3 presents summaries of both behavioral data and N170 amplitude data for both normal and dyslexic readers.

**Table 3:** Summary table of behavioral and EEG results.

		**HC vs.VC**	**HC vs. DC**	**DC vs. DE**	
Normal	ACC	> (0.93)	> (0.66)	<(0.51)	
	RT	< (0.15)	< (0.04)	ns	
	N170	> (0.03)	ns	> (0.53)	
		**HC**	**VC**	**DC**	**DE**
	Laterality	L> R (0.13)	L>R(1.6)	ns	ns
	Stimuli	Straight > curved			
Dyslexic	ACC	< (0.01)	< (0.05)	< (0.22)	
	RT	< (0.04)	> (0.04)	ns	
	N170	> (0.06)	ns	> (0.02)	
		**HC**	**VC**	**DC**	**DE**
	Laterality	ns	L>R(0.16)	ns	L>R(0.27)
	Stimuli	ns	ns	ns	Curved >straight

*Effect sizes represented by Cohen's d for the group comparison are reported in the parentheses. We calculated Cohen's d by using the following formula: [4*η*^2^/1-*η*^2^]^1/2^. Cohen's d < 0.2 indicates a small effect size, 0.2 < Cohen's d < 0.8 indicates a medium effect size, and Cohen's d > 0.8 indicates a large effect size (Fritz et al., 2012)*.

*VC, viewing character; HC, handwriting character; DC, drawing followed by Chinese recognition; DE, drawing followed by English recognition; ACC, accuracy rate for binary decision; RT, response time; L, left hemisphere; R, right hemisphere*.

## Discussion

We investigated handwriting in comparison to drawing on word recognition between typical and dyslexic readers. We first compared handwriting-Chinese (HC) with viewing-Chinese (VC) characters and with two other drawing conditions, i.e., drawing followed by Chinese recognition (DC) and drawing followed by English recognition (DE). Moreover, stimuli to be handwritten and drawn included both curved-line vs. straight-line strokes and curved-line vs. straight-line shapes. Five main findings were revealed. First, we found a Chinese handwriting facilitating effect in normal readers on behavior and the N170 compared to viewing in Chinese. Second, we found a drawing facilitating effect on English word recognition compared to Chinese character recognition for dyslexic readers, represented by behavioral and N170 indicators. Third, we revealed a laterality effect of handwriting in comparison to viewing Chinese among normal but not dyslexic readers, suggesting greater specialization in reading development in normal readers. Fourth, for normal readers, the left lateralization of the handwriting effects was supported by straight-line stimuli trials only but not for curved-line stroke handwriting. Finally, for dyslexic readers, the drawing effect on English word recognition was supported by curved-line shape drawing but not straight-line shape drawing.

The handwriting-Chinese condition (HC) has a facilitative effect on the Chinese word recognition when compared to the viewing-Chinese condition (VC), with longer reaction time and higher accuracy in VC than in HC. The peak N170 amplitude for HC in microvolts was likewise significantly larger than that of VC. Both the behavioral and ERP results show that HC facilitated the Chinese character processes, and when compared to the results for VC, HC facilitated the Chinese characters processing for typically developing but not dyslexic readers. The fact that the HC condition elicited a greater N170 than the VC condition indicates the N170 reflects efficient orthographic recognition due to handwriting experience, consistent with Liu and Perfetti's (2003) study results for Chinese-English bilinguals as well as other handwriting training (Guan et al., 2011, 2021) and Chinese word recognition research (Guan and Fraundorf, 2020; Guan et al., 2020). Handwriting training is hypothesized to improve recognition of the orthographic representation of the visual inputs in the human brain. Consistently, another study on artificial orthographies revealed that unit size gained during training impacted N170 modulation to word recognition (see Yoncheva et al., 2010). In current study, handwriting learning condition focused more on the smaller units embedded in the visual representation of the words. Participants had to evaluate if a single character was included exactly with the same form in a complicated compound character in a binary judgement. They paid attention to local features, which might facilitate early Chinese character processing online, thus causing the N170 modulation.

The handwriting effect, on the other hand, did not persist in the response patterns for the dyslexic readers in our study. For typically developing readers, handwriting practice, which focuses on the intricate visual-orthographic components of stroke construction, is expected to enhance motor-sensory integration to aid visual recognition (Guan et al., 2011, 2021). The lack of handwriting effects in the dyslexic readers might suggest that they could have trouble focusing on the intricate visual-orthographic components of strokes and configurations of the Chinese writing system. Accordingly, other findings have revealed that when past knowledge was controlled for, improvements in handwriting quality predicted advanced performance in reading (Guan et al., 2015). Thus, lacking progress in reading development might be related to the failure in handwriting practices. Handwriting provides a sources for sensory-motor integration in the native language, and then generates a mental representation in alignment with a neural motor memory in a newer and more solid manner, which helps to establish the reading framework in the brains of typically developing readers. Sensorimotor coding plays a facilitating role in language cognition (Guan and Wang, 2017). In other words, it is easier for those who have a better understanding of the visual-motor integration in this language to acquire the written language in a more refined manner of visual-motor coupling, thus producing a more robust visual-orthographic representation in the mental lexicon. Unfortunately, such sensory-motor training in the current study might be difficult for dyslexic readers to master with limited practice.

Moreover, a fMRI study suggested that Chinese dyslexic children showed abnormal brain activation in brain regions associated with motor and visual processing, as well as general executive control, during handwriting (Yang et al., 2021). Consequently, in addition to visual-motor integration processing, it is possible that handwriting recruits attentional resources. However, executive control is integral to the process of handwriting and its deficits. For instance, some studies have attributed the high rate of pauses during handwriting to orthographic spelling difficulties in dyslexia (Sumner et al., 2013, 2014), whereas others have suggested impairment of motor execution during handwriting in developmental dyslexia (DD) given that children with DD fail to comply with the principles of isochrony and homothety in the motor execution of handwriting (Pagliarini et al., 2015). Moreover, compared to age-matched and spelling-matched controls, people with DD are more impacted by the graphic complexity of words (Gosse and Van Reybroeck, 2020).

The accuracy level for HC was higher than DC, revealing that handwriting Chinese characters resulted in better performance than drawing followed by Chinese recognition, implying that handwriting facilitates in the coordination of the eye, mind and hands in order to establish a more sensible representation of the sub-lexicon forms (Guan et al., 2011). For readers who are typically developing, handwriting may help them perceive Chinese characters faster (Guan et al., 2015). For dyslexic readers only, however, DC reaction times were quicker than HC, and the EEG values for HC and DC were not statistically different. The findings suggested that both drawing could impact the N170, but handwriting might not. As a result, we will continue to investigate the stimuli effect in connection to the handwriting and drawing effect in both normal and dyslexic readers.

For typical readers, the lower accuracy rates for DC than HC suggested handwriting Chinese characters facilitated performance than drawing, and handwriting helped to coordinate the brain, eyes, and fingers to establish a subtle representation for sub-lexical word forms (Guan et al., 2011). Handwriting may accelerate the perception of Chinese characters for typically developing readers (Guan et al., 2015). However, the reaction times for DC were faster than HC for dyslexic readers only, and the ERP comparison between handwriting and drawing did not statistically differ from each other. The results suggest that a modulation in the ERPs indicator of the N170 by handwriting and drawing learning practices. Therefore, we continue examining the stimuli effect in correlation with the handwriting and drawing effect in typical and dyslexic readers.

The drawing followed by English and Chinese recognition differed between the DE and DC conditions. This may possibly reflect differences in the ways readers process Chinese and English. Above all, our results implied the language specificity effect. When processing Chinese, the brain functions the specific categorical perception in the written word unit. Therefore, processing Chinese characters might arouse a higher magnitude in N170 amplitude, and meanwhile the readers might show a more laterality in the left hemisphere in the N170 indicator. Previous study had a consistent findings in showing a more left-lateralized N170 in Chinese recodnition for English-Chinese bilinguals than the native-English readers (Wong et al., 2005). That is to say, the Chinese recognition, like processing faces could trigger a language-specific processing mechanism in the brain. Unfortunately, the laterality effect in the left hemisphere of this N170 to such language-specific stimuli is unclear.

Meanwhile, there was a drawing facilitation effect on the ERP indicator of N170 in the DE condition but not in the DC condition in dyslexic readers. But the results were vice versa for typically developing readers. This also shows a native language specificity effect. Chinese children children start to learn English in the grade 3. The stimuli in Chinese seemed to be more familiar than those in English stimuli to all participants in our study. Therefore, we found that normal students had a greater N170 magnitude on those Chinese stimuli than the English stimuli as English was the second language. This findings is in alignment with Liu and Perfetti's (2003). They found that the N170 effect on a native language in Chinese was larger than that an L2 language like English. Some other studies also showed that the N170 indexed visual-orthographic recognition processes. Stimuli in the orthographic stimuli (such as letter strings, non-words or real words) triggered a larger N170 effect than non-orthographic stimuli (such as shapes or other meaningless symbols) (Bentin et al., 1996; Pylkkanen and Marantz, 2003; Simon et al., 2004). Normal readers in our current study are much more familiar with their native language Chinese, so that there persisted greater N170 modulations by the native language if their reading networks develop well.

Typical readers showed a laterality effect on the N170 in the left hemisphere in the handwriting and viewing conditions. However there were no such laterality effect among the readers with developmental dyslexia. First, typical readers have had more experience with handwriting. They are not born with laterality according to literature, nor does the laterality effect appear in early stages of cognitive processes in children. As years grow, the laterality effect persists with written language when the readers mature (Kim et al., 2004). Consistent with the findings in Maurer et al. (2008), other studies have also reported an N170 facilitation for words in syllabic writing systems compared to the control (Shirahama et al., 2004). Shirahama et al. did not test left laterality effect, but this effect persisted among the experienced readers when they processed the alphabetic scripts (Bentin et al., 1996; Rossion et al., 2003; Maurer et al., 2005, 2008). Our findings echoed the same underlying mechanism of N170 laterality in the Chinese writing system that contained larger features of orthographic units, including syllables.

More importantly, our findings are consistent previous studies that demonstrated laterality effect in Chinese word recognition. Cao et al. (2011) claimed that the specialized mechanism of Chinese word recognition should merge among children turn to 7-year-old. Researchers examined different aged readers (ranging from 7 to adults) and found the left laterality effect on N170 modulation. On the contrary, children with dyslexia (*M*_age_ = 9.5 years) did not have such an effect.

The absence of handwriting effects in dyslexic readers might be due to the following three reasons. First, the priming strokes of the basic symbols in the handwriting condition included only curved vs. straight-line strokes. These simple straight-line or curved-line handwriting experiences might not elicit dyslexic readers' sensitivity to the positional hierarchy and internal structure of the constituent parts of the Chinese characters (Leong et al., 2000). Second, basic stroke symbols do not facilitate grapheme-phoneme connection among dyslexic readers, who have deficits in grapheme-phoneme connection in reading performance (Aravena et al., 2013, 2017). A recent study showed that grapheme-phoneme learning training failed to significantly contribute to reading outcomes in an unknown orthography in dyslexic readers. This finding suggests that, to conquer the difficulties of dyslexia, readers should target phonological and orthographic knowledge directly mapping onto the grapheme-phoneme-conversion process itself (Law et al., 2018). A third reason might be due to the lack of handwriting practice. We speculate that increasing the number of handwriting practice trials might lead to different patterns of handwriting effects on dyslexic readers' word recognition.

Our findings revealed drawing effects of curved-line shapes on word recognition in English. Drawing curved shapes such as hearts, moons and waves involved studying highly variable instances of a symbol, facilitating symbol categorization relative to grapheme-motor connection of Chinese characters, regardless of visual-motor production (Li and James, 2016). This symbol categorization might not be a basic requirement for word recognition in English and might not a deficit among Chinese dyslexic readers. In fact, in our behavioral measures of English word reading, there were no differences between normal and dyslexic readers, leading us to speculate that that Chinese dyslexic readers might not perform worse in English reading. Xue et al. (2019) found an increased and left-lateralized N170 response for regular characters compared to cursive characters that were less familiar. It is likely that handwriting straight-line regular characters might prompt a quicker word recognition in Chinese. For the dyslexic readers, however, it is possible that the amount of training was not sufficient for increasing the familiarity of the visual characters for the children in our study.

Chinese children who are diagnosed with developmental dyslexia tend to have difficulty in spelling and reading Chinese characters, as well as writing and dictation (Leong et al., 2000). Nevertheless, the previous literature did not indicate that children with developmental dyslexia have trouble in drawing. For children with developmental dyslexia, the drawing skills acquired in free manual practice may improve children's visual mapping ability, thereby improving the visual-recognition patterns of the sequential letter recognition in English (Lam et al., 2011). For example, Seyll and Content (2020) evaluated the effects of graphic motor programs in letter-like shape recognition by interfering with graphic motor activity. The results showed that impaired handwriting was less accurate than normal handwriting, suggesting that handwriting motor skills contribute to the construction of letter representations. It is likely that, for Chinese dyslexic readers, a better way to improve their visual recognition skills could be through drawing practice (Poon et al., 2010) or that cursive pattern recognition skills could be improved through motor training like drawing (Schwellnus et al., 2012), improving English reading ability in which Chinese developmental dyslexics may not necessarily show impairments.

There are some limitations deserving consideration for future research. First, as we used the same stimuli across groups, the difficulty level of our stimuli might not be the same for typical and dyslexic readers. Future research should consider varying stimulus difficulty levels across age among typical and dyslexic readers, as the critical period for handwriting might begin at age of 7 and end at about 10 years old. Second, as the participants only engaged in handwriting or drawing for a few seconds, the modest effects might be due to limited prime duration. If participants are exposed to the learning conditions for a longer time period, there might be more significant effects and larger effect sizes. Third, future research should consider the possible effects of attention mechanisms on visual inputs (such as curved vs. straight shapes) on dyslexic children's handwriting in relation to orthographic features of linguistic writing units. It is speculated that the visual-form areas in the brain might be less activated by curved letters in comparison to straight-line letters in English as the visual-motor integrative processing of curved and smooth shapes requires less cognitive effort than straight but sharp-angled shapes (Ose Askvik et al., 2020). Moreover, the aesthetics of curved and smooth shapes might be more highly valued by dyslexic readers and may be processed at the same speed and with the same visual span as the normal readers (O'Brien et al., 2005). Finally, future research should also examine fine-grained modulation features of ERPs before 170 ms post stimulus onset (Woodman, 2010), which might reveal an effect of handwriting on sensory processing (Pratt, 2011), word recognition (Hillyard et al., 1998), or visual discrimination (Vogel and Luck, 2000).

In conclusion, handwriting straight-line Chinese characters led to a larger N170 laterality effect in the left hemisphere and quicker behavioral responses than viewing Chinese characters and quicker behavioral response than drawing for typically developing readers. Drawing curved-line shapes produced better performance in word recognition in English for dyslexic readers. The visual-motor integration mechanism might be the key underlying mechanism. The word visual representation might be enhanced by the efficient integration between visual and motor areas of the brain. This is the basic requirement for Chinese word recognition. The laterality effect in the left hemisphere was shown in normal but not dyslexic readers. The finding that drawing curved lines/shapes might enhance word recognition in English deserves more detailed future research. Future research should vary in methodologies to examine whether and to what extent handwriting or drawing affects orthographic perception among Chinese and English bilinguals.

## Data Availability Statement

The raw data supporting the conclusions of this article will be made available by the authors, without undue reservation.

## Ethics Statement

The studies involving human participants were reviewed and approved by University of Science and Technology Beijing. Written informed consent to participate in this study was provided by the participants' legal guardian/next of kin.

## Author Contributions

CG and WM designed the study, collected and analyzed the data, and wrote the paper. YL analyzed the data. LM wrote the paper. All authors contributed to the article and approved the submitted version.

## Funding

This study was supported by the Fundamental Research Funds for the Central Universities at Beijing Language and Culture University (#20YJ020015), Beijing Social Science Key-level Grant (18YYA001), and China National Science Foundations (62077011) awarded to the CG.

## Conflict of Interest

The authors declare that the research was conducted in the absence of any commercial or financial relationships that could be construed as a potential conflict of interest.

## Publisher's Note

All claims expressed in this article are solely those of the authors and do not necessarily represent those of their affiliated organizations, or those of the publisher, the editors and the reviewers. Any product that may be evaluated in this article, or claim that may be made by its manufacturer, is not guaranteed or endorsed by the publisher.
